# Discoscopic Findings of High Signal Intensity Zones on Magnetic Resonance Imaging of Lumbar Intervertebral Discs

**DOI:** 10.1155/2014/245952

**Published:** 2014-05-21

**Authors:** Kosuke Sugiura, Ichiro Tonogai, Tetsuya Matsuura, Kosaku Higashino, Toshinori Sakai, Naoto Suzue, Daisuke Hamada, Tomohiro Goto, Yoichiro Takata, Toshihiko Nishisho, Yuichiro Goda, Ryosuke Sato, Kenji Kondo, Fumitake Tezuka, Kazuaki Mineta, Makoto Takeuchi, Mitsuhiko Takahashi, Hiroshi Egawa, Koichi Sairyo

**Affiliations:** Department of Orthopedics, The University of Tokushima, 3-18-15 Kuramoto, Tokushima 770-8503, Japan

## Abstract

A 32-year-old man underwent radiofrequency thermal annuloplasty (TA) with percutaneous endoscopic discectomy (PED) under local anesthesia for chronic low back pain. His diagnosis was discogenic pain with a high signal intensity zone (HIZ) in the posterior corner of the L4-5 disc. Flexion pain was sporadic, and steroid injection was given twice for severe pain. After the third episode of strong pain, PED and TA were conducted. The discoscope was inserted into the posterior annulus and revealed a migrated white nucleus pulposus which was stained blue. Then, after moving the discoscope to the site of the HIZ, a migrated slightly red nucleus pulposus was found, suggesting inflammation and/or new vessels penetrating the mass. After removing the fragment, the HIZ site was ablated by TA. To our knowledge, this is the first report of the discoscopic findings of HIZ of the lumbar intervertebral disc.

## 1. Introduction

Discogenic low back pain is among the pathologies of chronic low back pain. However, making an exact diagnosis can be difficult. Several studies have reported the importance of discography and provoked signs in the diagnosis of discogenic pain [1, 2]. According to Bini et al., discography remains the only functional test capable of showing, both morphologically and provocatively, the discs that are involved in the patient's clinical picture [3]. Due to its invasiveness, however, discography has not become a standard diagnostic procedure. Instead, the less-invasive modality of magnetic resonance imaging (MRI) is frequently used.

In 1992, Aprill and Bogduk [4] reported a strong correlation between the annular high signal intensity zone (HIZ) on T2-weighted MRI and positive provocative discography, stating that the HIZ is an effective marker for discogenic back pain. Similar clinical studies followed [5–7]. Chen et al. [6] reported a correlation between the HIZ and positive concordant pain on discography, and Peng et al. [8] and Dongfeng et al. [9] found inflammation and neovascularization on histological analysis of disc material in the HIZ. Physicians should therefore be aware of the significance of the HIZ when diagnosing discogenic pain.

Percutaneous endoscopic discectomy (PED) with radiofrequency thermal annuloplasty (TA) is a minimally invasive surgical procedure for discogenic pain which originated from the techniques of Hijikata [10] and Kambin and Schaffer [11]. Establishment of this single-portal endoscopic discectomy was made possible by the great efforts of Yeung et al. [12–14]. PED with TA is minimally invasive and can be performed under local anesthesia via a 7 mm skin incision, and it provides the advantage of direct visualization of the degenerated disc and annular tear by means of a spinal endoscope. Under clear visualization of the endoscope, a degenerated nucleus pulposus (NP) can be removed. Furthermore, radiofrequency TA can be performed while viewing the annular tear endoscopically. The combined technique of PED and TA has been reported to be effective for discogenic back pain [15–17]. In this paper, we report a case of chronic low back pain with an HIZ treated by applying the PED and TA technique. Furthermore, we demonstrate for the first time the discoscopic findings of the HIZ site obtained with a spinal endoscope.

## 2. Case Report

A 32-year-old man, who played nonprofessional baseball, consulted our hospital for chronic low back pain. Within the last year he had experienced a severe episode of pain during flexion, for which he was successfully treated with an intradiscal steroid injection. His diagnosis was discogenic pain with an HIZ in the posterior corner of the L4-5 disc. After experiencing a second episode of severe low back pain during flexion, he was referred to our hospital. He complained of low back pain during lumbar flexion and slight pain during extension. However, no leg symptoms were present and all neurological signs were normal.

**Figure 1:**
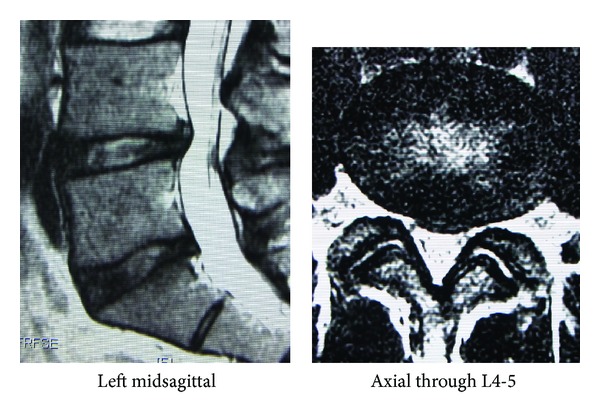
T2-weighted magnetic resonance imaging (MRI) findings on the initial visit. A degenerated disc with a slight protrusion is visible; however, no high signal intensity zone (HIZ) is obvious.

**Figure 2:**
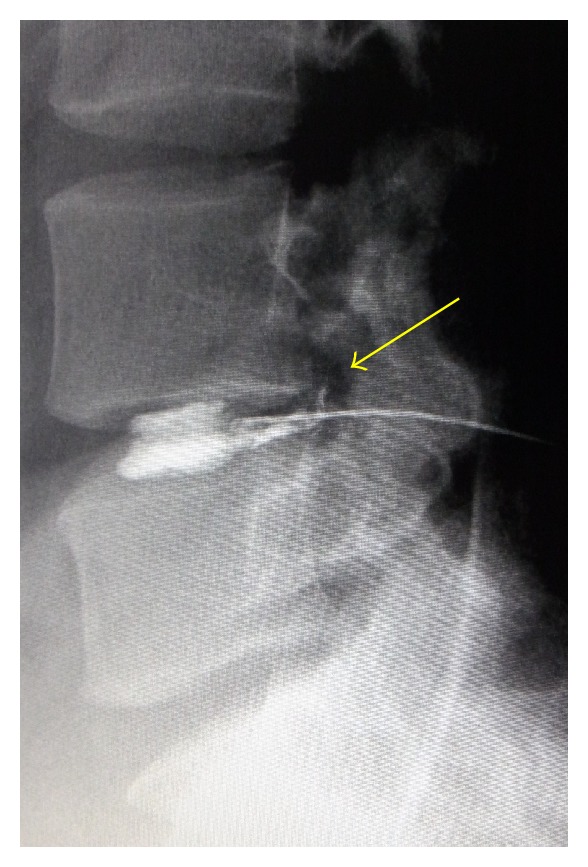
Discography findings showing leakage of contrast media into the annular tear. At this time, the patient reported concordant low back pain, which was completely relieved by intradiscal injection.

Figure 1 shows the MRI findings just after the second episode. The disc showed degeneration with a slight protrusion; however, the HIZ was not so obvious. We conducted discography and administered a steroid injection at the disc (Figure 2). Discography revealed contrast media leakage into the annular tear. At this time, the patient reported concordant low back pain, which was completely relieved by the intradiscal injection. The patient then returned to baseball practice. During a practice session at spring camp, 2 weeks after the second injection, he again experienced mild discomfort in his back. The next day, he could not move because of severe pain, so he took analgesic medicine. However, as no pain relief was obtained for a week, he visited us again.

**Figure 3:**
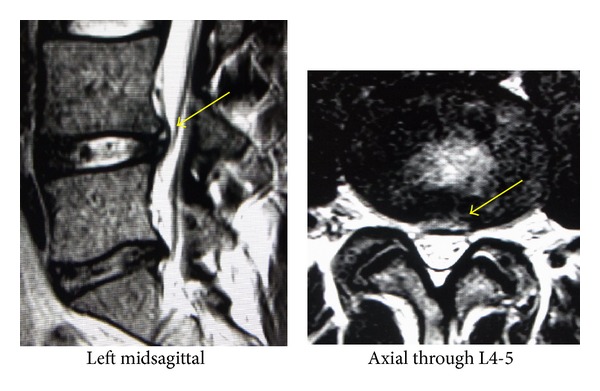
T2-weighted MRI findings on the second visit. Sagittal and axial images both show that the size of the disc protrusion is similar to that in the first MRI. This time, however, the HIZ is obvious.

Figure 3 shows the MRI findings just after the third episode of severe low back pain. The sagittal and axial images both showed that the size of the disc protrusion was similar to that on the first MRI, with the exception of the obvious HIZ. As this was the third episode, we decided to perform minimally invasive endoscopic surgery with PED and TA, instead of the previous conservative care.

Surgery was conducted on the basis of a review article on the PED procedure [18]. For the present case, the transforaminal approach was selected and an 8 mm lateral skin incision was made about 8 cm from the midline. After providing sufficient local anesthesia around the disc, a needle was inserted into the disc through the safety triangle. Next, discography was conducted with indigo carmine to dye the NP and the displaced fragment blue. A guide pin was inserted into the disc through the puncture needle, and the obturator and cannula were inserted sequentially. After inserting the cannula, PED and TA were initiated.

**Figure 4:**
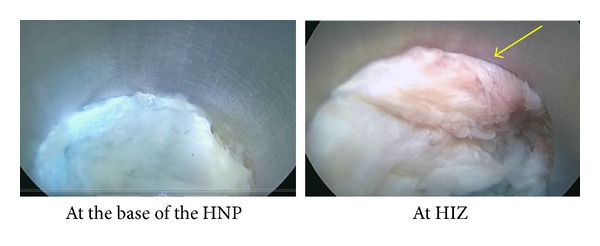
Endoscopic view of the disc showing the migrated white nucleus fibrosus (left panel). At the site showing the HIZ, a slightly red migrated nucleus pulposus is apparent (right panel), suggesting inflammation and/or penetration of new vessels into the mass.

**Figure 5:**
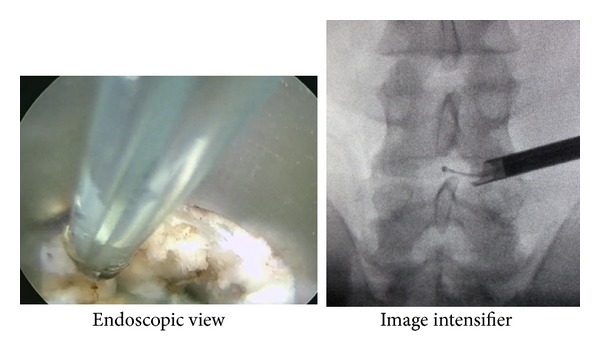
Radiofrequency thermal annuloplasty.

During surgery, the scope was inserted into the posterior annulus where it revealed a migrated NP dyed blue (Figure 4, left panel). After removing the displaced NP, the annular tear was clearly evident due to being filled with the blue NP. When the scope was moved to the site of the HIZ through the annular tear, a slightly red migrated NP was found (Figure 4, right panel), suggesting inflammation and/or new vessels penetrating the mass. After removing the slightly red migrated NP, the HIZ site was ablated with a radiofrequency coagulator (i.e., TA) (Figure 5).

**Figure 6:**
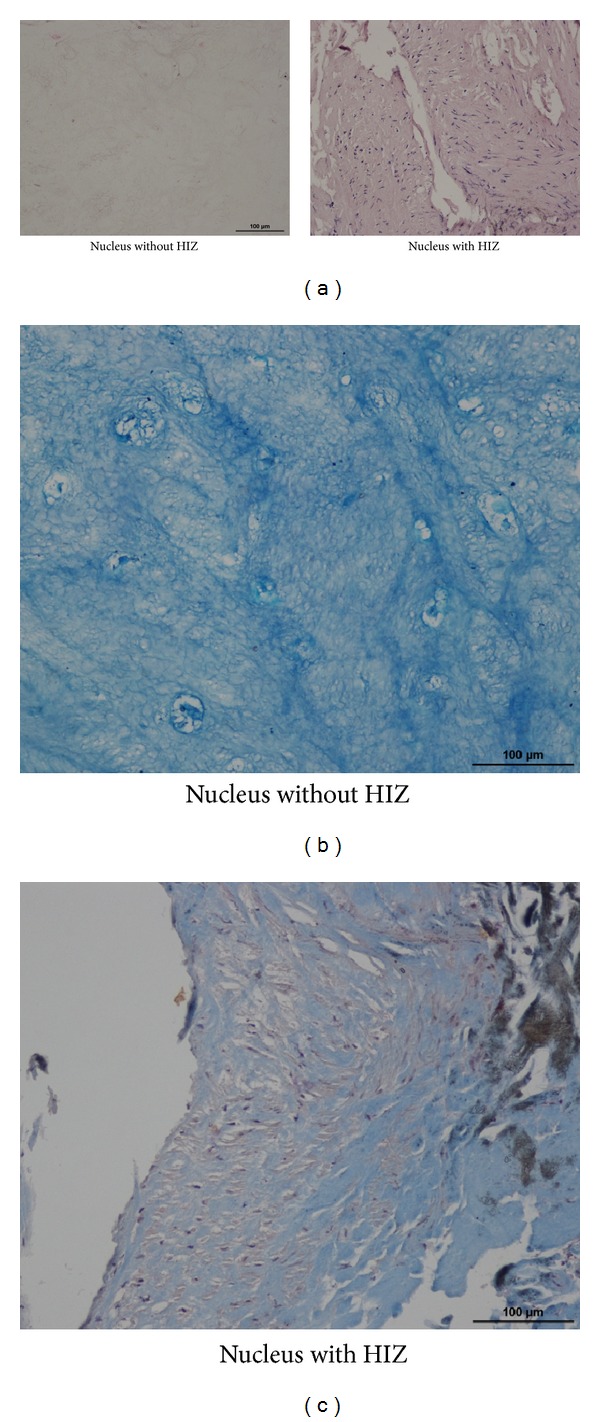
Histological findings of two kinds of tissue: the displaced nucleus pulposus (NP) with (right panel) and without (left panel) an HIZ. (a) Hematoxylin and eosin staining showing the NP without an HIZ filled with cartilaginous tissue. (b) Alcian blue staining showing the NP without an HIZ consisting of extracellular matrix of proteoglycan-based cartilage. (c) Masson trichrome staining showing the NP with the HIZ containing many fibroblastic cells, not chondrocytes, as well as a fibrotic matrix.

Figure 6 shows the histological findings of two kinds of tissue, the displaced NP with (right panel) and without (left panel) an HIZ. On hematoxylin and eosin staining, the NP without the HIZ was found to be filled with cartilaginous tissue, with an extracellular matrix consisting of proteoglycan-based cartilage (alcian blue staining). On the other hand, the migrated mass with the HIZ contained many fibroblast-like cells, not chondrocytes, and the matrix was fibrotic (Masson trichrome staining).

Within 6 weeks of surgery, the severe low back pain during lumbar flexion had resolved, although slight pain during extension persisted. The patient initiated trunk muscle isometric training and stretching, and walking and jogging were allowed as light exercise.

## 3. Discussion

Diagnosis of discogenic pain is sometimes difficult. Provocative discography has been reported to be effective for its diagnosis, but the technique is controversial due to its invasiveness [1, 2]. Instead, various MRI indicators have been investigated, and among them the HIZ has recently been reported by many reports to be an effective indicator for discogenic pain [4–9]. However, the significance of the HIZ remains to be established [9].

In 1992, Aprill and Bogduk [4] reported a strong correlation between annular HIZ on T2-weighted MRI and positive provocative discography. Chen et al. [6] reported a correlation between the HIZ and positive concordant pain during discography. Kim et al. [19] analyzed the MRI findings of several cases of acute severe low back pain and found an HIZ in 61% of cases, emphasizing the importance of the HIZ in understanding discogenic pain. Wang and Hu [20] retrospectively reviewed 3,115 discs on 623 lumbar MR images to understand the meaning of the HIZ in cases of back pain. The majority of the HIZ was at the L4-5 or L5-S level, and 57.5% of the HIZ-positive patients were symptomatic. Although some researchers do not consider HIZ to be related to low back pain, many clinical studies and histological investigations have shown that the HIZ has the potential to be a good indicator for discogenic low back pain [9].

In 2006, Peng et al. [8] sampled the disc materials from the HIZ site during interbody fusion surgery and subjected them to histological analysis. Findings revealed the formation of vascularized granulation tissue in the outer region of the annulus fibrosus, suggesting that the HIZ can be a good indicator for discogenic low back pain. More recently in 2011, Dongfeng et al. [9] evaluated inflammatory reactions at the HIZ site using an immunohistochemical technique. They analyzed disc tissue samples taken from 26 patients during lumbar interbody fusion surgery and found that the disc tissue with an HIZ had a proliferation of small round cells and fibroblasts. In addition, an abundance of tumor necrosis factor-alpha-positive cells and some CD68-positive cells was observed in the HIZ. They therefore concluded that the HIZ can serve as a sign of disc pain.

In this study, by means of a spinal endoscope (i.e., discoscopy), we observed the intradiscal site showing an HIZ on MRI. As shown in Figure 4, the white displaced NP with posterior annulus fibrosus without an HIZ was stained blue with indigo carmine. On the other hand, the displaced NP with the HIZ was slightly red, suggesting inflammation in the displaced NP. Histological findings showed that the displaced mass with the HIZ contained many fibroblast-like cells and the matrix was fibrotic. This is in good agreement with previous histological studies on the HIZ [8, 9].

Since Carragee et al.'s report in 2009 [21], the role of discography has been controversial [17, 21–23], because discography was suggested to actually accelerate disc degeneration. Recently, Ohtori et al. [23] followed patients who underwent discography for 5 years and found no significant differences in the rate of disc degeneration compared with the control group. Moreover, X-ray images showed no significant differences in disc height, range of motion, or translation between the flex and extension position (*P* > 0.1). Thus, they concluded that discography does not accelerate disc degeneration, at least within the first 5 years. Furthermore, discography and provoked signs have been reported to be important for the diagnosis of discogenic pain [17]. Bini et al. stated that discography remains the only functional test that can show both morphologically and provocatively which discs are involved in the patient's clinical picture. Our institution has been conducting discography on selected patients based on our findings of a literature review.

To date, few studies have provided endoscopic and discoscopic images of the HIZ site on MRI. We anticipate that further application of the PED system in this kind of imaging study will lead to progress in the treatment and diagnosis of discogenic pain.
